# 2-Amino-4-(4-chloro-1-ethyl-2,2-dioxo-1*H*-benzo[*c*][1,2]thia­zin-3-yl)-7,7-dimethyl-5-oxo-5,6,7,8-tetra­hydro-4*H*-chromene-3-carbo­nitrile: single-crystal X-ray diffraction study and Hirshfeld surface analysis

**DOI:** 10.1107/S2056989021002085

**Published:** 2021-02-26

**Authors:** Mariia O. Shyshkina, Dmitry A. Lega, Volodymyr D. Goryachiy, Ludmila M. Shemchuk, Dmitriy V. Levashov, Leonid A. Shemchuk

**Affiliations:** a SSI Institute for Single Crystals NAS of Ukraine, 60 Nauky ave., Kharkiv 61001, Ukraine; b National University of Pharmacy, 4 Valentynivska st., Kharkiv 61168, Ukraine

**Keywords:** 1*H*-benzo[*c*][1,2]thia­zine, 2,2-dioxide, 4*H*-pyran, multicomponent reaction, mol­ecular structure, crystal structure, Hirshfeld surface analysis

## Abstract

In the title compound, C_22_H_22_ClN_3_O_4_S, which has potential non-steroidal anti-inflammatory activity, the benzo­thia­zine and cyclo­hexenone rings both adopt a distorted sofa conformation while the 4*H*-pyrane ring adopts a very flattened sofa conformation. The two bicyclic fragments are skewed to each other, with the dihedral angle between their least-squares planes being 72.8 (1)°. In the crystal, the mol­ecules form a hydrogen-bonded chain parallel to the *a* axis due to N—H⋯O and N—H⋯Cl hydrogen bonds. Neighbouring chains are linked by C—H⋯N, C—H⋯O and π–π stacking inter­actions. Hirshfeld surface analysis was used to investigate the importance of the different types of inter­molecular inter­actions whose contributions are: H⋯H = 44.7%, O⋯H/H⋯O = 21.8%, N⋯H/H⋯N = 11.9%, C⋯H/H⋯C = 9.5%, Cl⋯H/H⋯Cl = 7.2%. Parts of the mol­ecule, *viz*. the phenyl ring and the ethyl side chain, are equally disordered over two sets of sites.

## Abstract 2

The mol­ecular and crystal structures were studied and a Hirshfeld surface analysis undertaken for the title benzo­thia­zine derivative, which has potential non-steroidal anti-inflammatory activity.

## Chemical context   

The 1*H*-benzo[*c*][1,2]thia­zine 2,2-dioxide moiety and its derivatives have been the focus of chemists and pharmacologists for decades (Catsoulacos & Camoutsis, 1979 ▸; Ukrainets *et al.*, 2014 ▸; Iwatani *et al.*, 2013 ▸). These compounds have also gained additional value from a structural point of view because they can be regarded as bioisosteres of the 2,3-di­hydro-4*H*-benzo[*e*][1,2]thia­zin-4-one 1,1-dioxide core, which is a motif of well-known non-steroidal anti-inflammatory drugs (NSAIDs) of the ‘oxicame’ group (Lega *et al.*, 2016**b* ▸)*.

While synthesizing new mol­ecules, researchers often combine the 1*H*-benzo[*c*][1,2]thia­zine 2,2-dioxide core with other pharmacophores of a heterocyclic nature (Tomita *et al.*, 2013 ▸; Popov *et al.*, 2010 ▸; Cecchetti *et al.*, 1982 ▸). Recently, we have reported a series of compounds comprising a condensed system of 1*H*-benzo[*c*][1,2]thia­zine 2,2-dioxide and 2-amino-4*H*-pyran fragments (Lega *et al.*, 2017 ▸; Shemchuk *et al.*, 2014 ▸). The pronounced analgesic and anti-inflammatory properties of the products have also been confirmed (Lega *et al.*, 2016*a* ▸).

**Figure 1:**
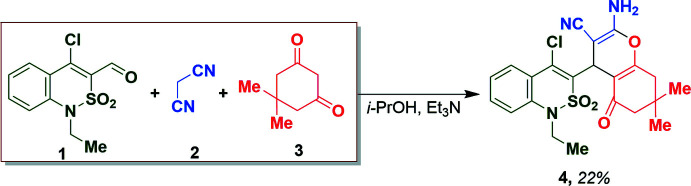
Synthesis scheme of the title compound **4**.

A three-component reaction of 4-chloro-1-ethyl-1*H*-benzo[*c*][1,2]thia­zin-3-carbaldehyde 2,2-dioxide, malono­nitrile and 5,5-di­methyl­cyclo­hexane-1,3-dione resulted in a new heterocyclic compound comprising σ-linked benzo[*c*][1,2]thia­zine 2,2-dioxide and 2-amino-4*H*-pyran moieties (Fig. 1 ▸). Under consideration of all the above-mentioned points, the product of the reaction as well as similar structures are potential bioactive substances, particularly with regard to NSAID activity. In this context, the mol­ecular and crystal structures were determined and a Hirshfeld surface analysis undertaken for the title compound, **4**.
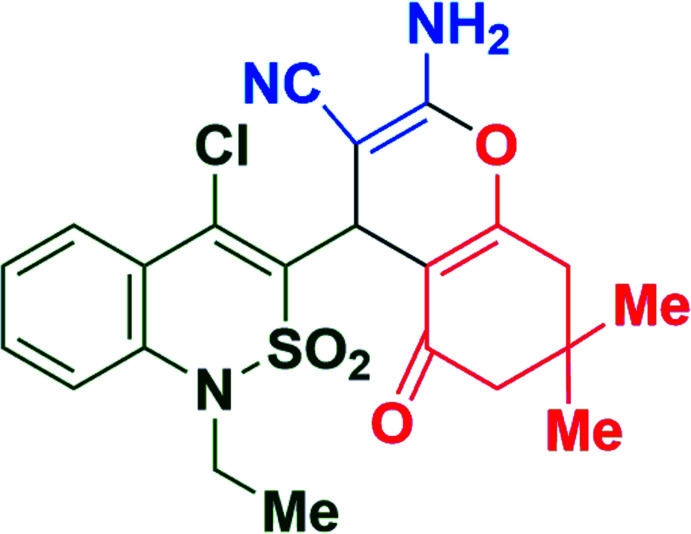


## Structural commentary   

**Figure 2:**
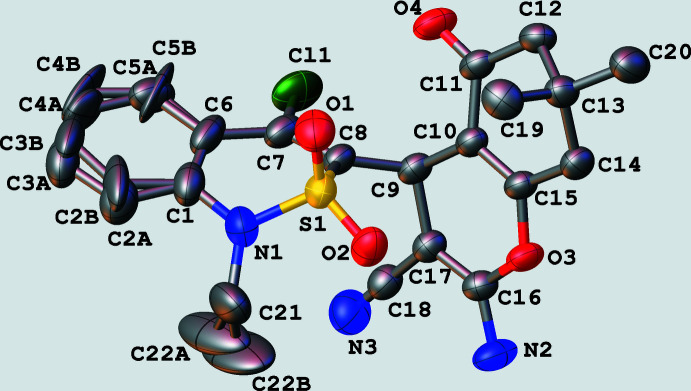
The mol­ecular structure of compound **4**. Displacement ellipsoids are drawn at the 50% probability level.

The di­hydro­thia­zine ring of compound **4** adopts a distorted sofa conformation (Fig. 2 ▸) with puckering parameters (Zefirov *et al.*, 1990 ▸) of *S* = 0.63 (1), Θ = 52.5 (1)°, Ψ = 20.3 (1)°. The S1 and C8 atoms deviate from the least-squares plane of the remaining atoms of the ring by 0.863 (6) and 0.244 (2) Å respectively. The phenyl ring of the benzo­thia­zine fragment is disordered over two positions (*A* and *B*) with equal occupancy. The partially saturated carbocycle has the same conformation as the hydro­thia­zine ring, with puckering parameters of *S* = 0.67 (1), Θ = 41.9 (1)°, Ψ = 11.8 (1)°. The deviations of the C13 and C14 atoms from the least-squares plane of the remaining atoms in the ring are 0.717 (2) and 0.132 (2) Å, respectively. The 4*H*-pyran ring adopts a very flattened sofa conformation with puckering parameters of *S* = 0.11 (1), Θ = 59.3 (1)°, Ψ = 3.2 (1)°, where the C9 atom deviates by 0.118 (2) Å from the plane of the remaining atoms in this ring. The C8—C9 bond is elongated to 1.525 (3) Å [the mean value (Orpen *et al.*, 1994 ▸) for a C*sp* ^2^—C*sp* ^3^ bond is 1.510 Å] to compensate for the steric repulsion between the bicyclic fragments. The bicycles are skewed in relation to each other [the dihedral angle between their mean planes is 72.8 (1)°]. The presence of the vicinal substituents on the 4*H*-pyran moiety results in an elongation of the C16—C17 bond to 1.347 (3) Å [the mean value for the C*sp* ^2^—C*sp* ^2^ bond is 1.331 Å; Orpen *et al.*, 1994 ▸] due to steric repulsion between them; the H2*B*⋯C18 distance is 2.57 (3) Å compared to the van der Waals radii sum (Zefirov, 1997 ▸) of 2.87 Å. The C21—C22 bond is located in a *syn*-clinal position to the C1—N1 endocyclic bond and the C22 atom is disordered over two positions (*A* and B*)* with equal occupancy due to rotation around the N1—C21 bond [the C22*A*—C21—N1—C1 torsion angle is 56.8 (9)° while the C22*B*—C21—N1—C1 torsion angle is 77.0 (11)°].

## Supra­molecular features   

**Figure 3:**
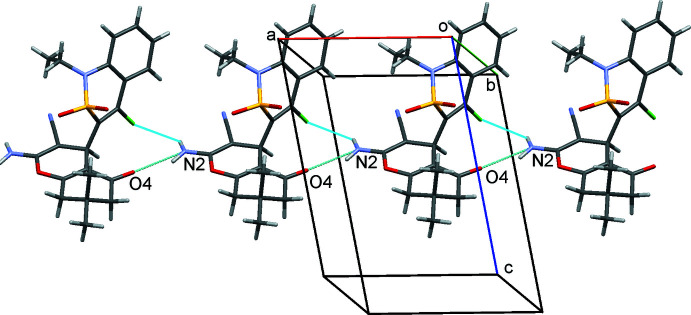
The chain of mol­ecules **4** linked through N—H⋯O and N—H⋯Cl hydrogen bonds.

In the crystal, mol­ecules of **4** form hydrogen-bonded chains parallel to the *a* axis (Fig. 3 ▸) due to N2—H2*A*⋯O4^i^ and N2—H2*B*⋯Cl1^i^ inter­molecular inter­actions [symmetry code: (i) 1 + *x*, *y*, *z*; Table 1 ▸]. Stacking inter­actions between di­hydro­thia­zine fragments of neighbouring chains occur [the distance between di­hydro­thia­zine rings is 3.77 (1) Å, the plane shift is 3.198 (1) Å]. As a result, layers parallel to (011) may be considered as secondary structural motifs.

Further stacking inter­actions between 4*H*-pyran rings of mol­ecules belonging to neighbouring layers are found [the distance between ring planes is 3.38 (1) Å and the plane shift is 1.247 (1) Å]. Mol­ecules are arranged in a head-to-tail manner in both types of stacking dimers. Additional C—H⋯N and C—H⋯O hydrogen-bonding inter­actions of a weak nature (Table 1 ▸) consolidate the packing of the mol­ecules in the crystal structure.

## Hirshfeld surface analysis   

**Figure 4:**
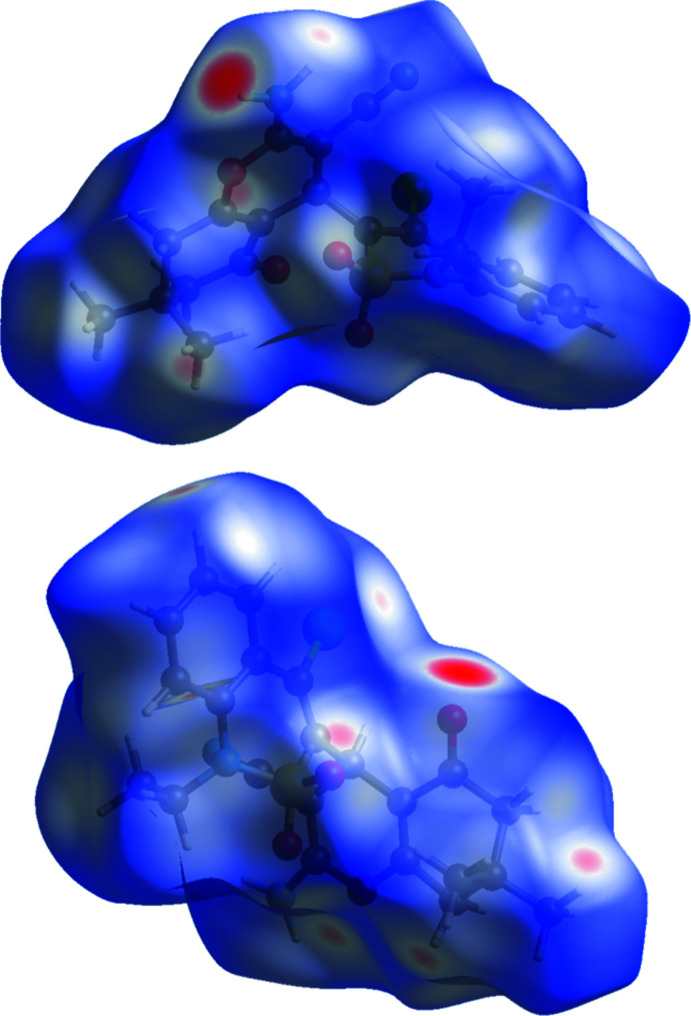
Two views of the Hirshfeld surface of mol­ecule **4** mapped over *d*_norm_ in the range −0.495 to 1.558 a.u.

Hirshfeld surface analysis (Turner *et al.*, 2017 ▸) was used to identify and visualize different types of intra- and inter­molecular inter­actions in the crystal structure. The mol­ecular Hirshfeld surface of the title compound was constructed using a standard (high) surface resolution with three-dimensional *d*_norm_ surfaces. The areas coloured red on the *d*_norm_ surfaces correspond to contacts that are shorter than the van der Waals radii sum of the closest atoms (Fig. 4 ▸). Red spots on the Hirshfeld surface indicate atoms participating in hydrogen bonding or short contacts. The brightest red spots are observed at one of hydrogen atoms of the amino group and at the carbonyl oxygen atom of the cyclo­hexenone fragment, indicating the strong inter­molecular N—H⋯O hydrogen bond. The smaller red areas are found at the other hydrogen atom of the amino group and the chlorine atom that indicates the N—H⋯Cl hydrogen bond. In addition, small spots are present at some of hydrogen atoms, as well as at the pyrane oxygen atom.

**Figure 5:**
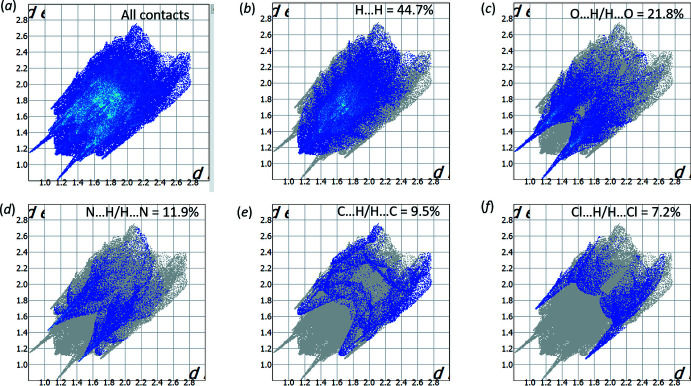
Two-dimensional fingerprint plot for compound **4** showing (*a*) all inter­actions, and delineated into (*b*) H⋯H, (*c*) O⋯H/ H⋯O, (*d*) N⋯H/H⋯O, (*e*) C⋯H/H⋯C and (*f*) Cl⋯H/H⋯Cl contacts.

All of the hydrogen bonds and short contacts of the title compound are evident on the two-dimensional fingerprint plot presented in Fig. 5 ▸ *a*. The pair of sharp spikes indicates the presence of strong hydrogen bonds in the crystal structure. The main contribution with respect to these spikes (21.8%) is provided by O⋯H/H⋯O inter­actions (Fig. 5 ▸ *c*), while the highest contribution is from H⋯H contacts (44.7%). The contributions of N⋯H/H⋯N (11.9%), C⋯H/H⋯C (9.5%) and Cl⋯H/H⋯Cl (7.2%) (Fig. 5 ▸ *d*, 5*e*, 5*f*) inter­actions are similar, but the presence of sharp spikes on the fingerprint plot containing only N⋯H/H⋯N or Cl⋯H/H⋯Cl inter­actions suggests that the latter contacts are much stronger.

## Database survey   

A search of the Cambridge Structural Database (CSD Version 5.41, update of November 2019; Groom *et al.*, 2016 ▸) for the benzo­thia­zine fragment revealed 44 hits. However, a chloro-substituted derivative was not found among these structures. It should be noted that the conformation of the benzo­thia­zine ring and redistribution of the electron density in the title compound is very similar to those found in the structures containing a methyl group or a hydrogen atom instead of the chlorine substituent [refcodes: KEGNAO (Nguyen & Retailleau, 2017 ▸), KESJEA (Ghandi *et al.*, 2014*a* ▸), OWUQII (Azotla-Cruz *et al.*, 2016 ▸), POJHUU, POJJAC, POJJEG, POJJIK, POJJOQ, POJJUW, POJKAD, POJKEH (Ukrainets *et al.*, 2018 ▸), ROJNOV (Ghandi *et al.*, 2014*b* ▸), VAZQEV, VAZQIZ (Azotla-Cruz *et al.*, 2017 ▸), ZIJQER (Shishkina *et al.*, 2018 ▸)].

The bicyclic fragment containing 4*H*-pyrane, cycloxenenone as well as amino and cyano substituents is found in 102 hits extracted from the CSD. In all of these structures, the conformation of this fragment is similar.

## Synthesis and crystallization   

A mixture of 4-chloro-1-ethyl-1*H*-benzo[*c*][1,2]thia­zin-3-carbaldehyde 2,2-dioxide (**1**) (0.271 g, 0.01 mol), malono­nitrile (**2**) (0.066 g, 0.01 mol) and 5,5-di­methyl­cyclo­hexane-1,3-dione (**3**) (0.140 g, 0.01 mol) was dissolved in 20 ml of *i*-PrOH and then tri­ethyl­amine (0.1 mol%) was added (Fig. 1 ▸). The mixture was refluxed for 4 h, then cooled to room temperature and left for an1 h. The resulting precipitate of compound **4** was filtered off, washed with *i-*PrOH, dried in air and recrystallized from *i-*PrOH. Yield 0.101 g (22%); colourless crystals; m.p. > 523 K.

## Refinement   

Crystal data, data collection and structure refinement details are summarized in Table 2 ▸. The bond lengths in the two disordered fragments were modelled with fixed values (C*sp* ^3^—C*sp* ^3^ = 1.54 Å for the ethyl side chain C21—C22; C*sp* ^2^—C*sp* ^2^ = 1.38 Å for the phenyl ring C1–C6), and with an equal occupancy for the two sets of sites. All hydrogen atoms were located in difference-Fourier maps. They were included in calculated positions and treated as riding with C—H = 0.96 Å, *U*_iso_(H) = 1.5*U*_eq_(C) for methyl groups and with Car—H = 0.93 Å, C*sp* ^3^—H = 0.97 Å, *U*_iso_(H) = 1.2*U*_eq_(C) for all other hydrogen atoms. The hydrogen atoms of the amino group were refined freely.

## Supplementary Material

Crystal structure: contains datablock(s) I. DOI: 10.1107/S2056989021002085/wm5596sup1.cif

Structure factors: contains datablock(s) I. DOI: 10.1107/S2056989021002085/wm5596Isup2.hkl

Supporting information file. DOI: 10.1107/S2056989021002085/wm5596Isup3.cml

CCDC reference: 2064493

Additional supporting information:  crystallographic information; 3D view; checkCIF report

## Figures and Tables

**Table 1:** Hydrogen-bond geometry (Å, °)

*D*—H⋯*A*	*D*—H	H⋯*A*	*D*⋯*A*	*D*—H⋯*A*
N2—H2*A*⋯O4^i^	0.92 (3)	2.03 (3)	2.857 (3)	150 (3)
N2—H2*B*⋯Cl1^i^	0.91 (3)	2.84 (3)	3.583 (3)	140 (2)
C4*B*—H4*B*⋯N3^ii^	0.93	2.49	3.381 (18)	160
C19—H19*C*⋯O3^iii^	0.96	2.56	3.452 (3)	155
C20—H20*C*⋯O1^iv^	0.96	2.56	3.443 (4)	153

Symmetry codes: (i) x+1, y, z; (ii) -x, -y+1, -z; (iii) -x+1, -y, -z+1; (iv) -x, -y, -z+1.

**Table 2:** Experimental details

Crystal data	
Chemical formula	C_22_H_22_ClN_3_O_4_S
*M*_r_	459.93
Crystal system, space group	Triclinic, *P*\overline{1}
Temperature (K)	293
*a*, *b*, *c* (Å)	8.6739 (5), 10.5490 (5), 12.4021 (8)
α, β, γ (°)	91.351 (4), 101.065 (5), 97.235 (4)
*V* (Å^3^)	1103.51 (11)
*Z*	2
Radiation type	Mo *K*α
μ (mm^−1^)	0.30
Crystal size (mm)	0.20 × 0.20 × 0.15

Data collection	
Diffractometer	Rigaku Oxford Diffraction Xcalibur, Sapphire3
Absorption correction	Multi-scan (*CrysAlis PRO*; Rigaku OD, 2018 ▸)
*T*_min_, *T*_max_	0.495, 1.000
No. of measured, independent and observed [*I* > 2σ(*I*)] reflections	11902, 6933, 3942
*R*_int_	0.057
(sin θ/λ)_max_ (Å^−1^)	0.754

Refinement	
*R*[*F* ^2^ > 2σ(*F* ^2^)], *wR*(*F* ^2^), *S*	0.073, 0.232, 1.00
No. of reflections	6933
No. of parameters	337
No. of restraints	12
H-atom treatment	H atoms treated by a mixture of independent and constrained refinement
Δρ_max_, Δρ_min_ (e Å^−3^)	0.33, −0.72

Computer programs: *CrysAlis PRO* (Rigaku OD, 2018 ▸), *SHELXT2014/5* (Sheldrick, 2015*a* ▸), *SHELXL2016/6* (Sheldrick, 2015*b* ▸), *Mercury* (Macrae *et al.*, 2020 ▸) and *publCIF* (Westrip, 2010 ▸).

## supplementary crystallographic information

### Crystal data

**Table:**

C_22_H_22_ClN_3_O_4_S	*Z* = 2
*M**_r_* = 459.93	*F*(000) = 480
Triclinic, *P*1	*D*_x_ = 1.384 Mg m^−^^3^
*a* = 8.6739 (5) Å	Mo *K*α radiation, λ = 0.71073 Å
*b* = 10.5490 (5) Å	Cell parameters from 2322 reflections
*c* = 12.4021 (8) Å	θ = 3.6–29.8°
α = 91.351 (4)°	µ = 0.30 mm^−^^1^
β = 101.065 (5)°	*T* = 293 K
γ = 97.235 (4)°	Block, colourless
*V* = 1103.51 (11) Å^3^	0.20 × 0.20 × 0.15 mm

### Data collection

**Table:**

Rigaku Oxford Diffraction Xcalibur, Sapphire3 diffractometer	6933 independent reflections
Radiation source: Enhance (Mo) X-ray Source	3942 reflections with *I* > 2σ(*I*)
Detector resolution: 16.1827 pixels mm^-1^	*R*_int_ = 0.057
ω scans	θ_max_ = 32.4°, θ_min_ = 3.2°
Absorption correction: multi-scan (CrysAlisPro; Rigaku OD, 2018)	*h* = −12→11
*T*_min_ = 0.495, *T*_max_ = 1.000	*k* = −15→15
11902 measured reflections	*l* = −18→18

### Refinement

**Table:**

Refinement on *F*^2^	12 restraints
Least-squares matrix: full	Hydrogen site location: mixed
*R*[*F*^2^ > 2σ(*F*^2^)] = 0.073	H atoms treated by a mixture of independent and constrained refinement
*wR*(*F*^2^) = 0.232	*w* = 1/[σ^2^(*F*_o_^2^) + (0.1119*P*)^2^] where *P* = (*F*_o_^2^ + 2*F*_c_^2^)/3
*S* = 1.00	(Δ/σ)_max_ < 0.001
6933 reflections	Δρ_max_ = 0.33 e Å^−^^3^
337 parameters	Δρ_min_ = −0.72 e Å^−^^3^

### Special details

**Table:**

Geometry. All esds (except the esd in the dihedral angle between two l.s. planes) are estimated using the full covariance matrix. The cell esds are taken into account individually in the estimation of esds in distances, angles and torsion angles; correlations between esds in cell parameters are only used when they are defined by crystal symmetry. An approximate (isotropic) treatment of cell esds is used for estimating esds involving l.s. planes.

### Fractional atomic coordinates and isotropic or equivalent isotropic displacement parameters (Å^2^)

**Table:**

	*x*	*y*	*z*	*U*_iso_*/*U*_eq_	Occ. (<1)
Cl1	0.03972 (9)	0.51417 (8)	0.26644 (8)	0.0788 (3)	
S1	0.20928 (7)	0.15439 (6)	0.25028 (5)	0.04761 (19)	
O1	0.0808 (2)	0.07168 (18)	0.27577 (16)	0.0648 (5)	
O2	0.3663 (2)	0.12600 (17)	0.29009 (15)	0.0575 (5)	
O3	0.58348 (18)	0.26894 (16)	0.50631 (14)	0.0477 (4)	
O4	0.0502 (2)	0.31102 (17)	0.50362 (17)	0.0592 (5)	
N1	0.1851 (3)	0.1610 (2)	0.11726 (17)	0.0620 (6)	
N2	0.7433 (3)	0.3725 (2)	0.4084 (2)	0.0616 (6)	
H2A	0.821 (4)	0.340 (3)	0.456 (2)	0.064 (8)*	
H2B	0.772 (4)	0.429 (3)	0.359 (3)	0.065 (9)*	
N3	0.5170 (3)	0.5555 (2)	0.2211 (2)	0.0720 (7)	
C1	0.0509 (4)	0.2134 (3)	0.0640 (2)	0.0685 (8)	
C2A	−0.0047 (19)	0.1696 (12)	−0.0450 (6)	0.075 (3)	0.5
H2AA	0.046249	0.110365	−0.076725	0.090*	0.5
C3A	−0.1356 (18)	0.2143 (15)	−0.1057 (12)	0.081 (4)	0.5
H3A	−0.177591	0.188100	−0.178732	0.097*	0.5
C4A	−0.198 (2)	0.302 (2)	−0.0480 (12)	0.085 (3)	0.5
H4A	−0.291494	0.328402	−0.084953	0.102*	0.5
C5A	−0.1415 (12)	0.3545 (14)	0.0572 (9)	0.079 (4)	0.5
H5A	−0.190299	0.416714	0.086940	0.095*	0.5
C2B	−0.0394 (19)	0.1748 (17)	−0.0399 (7)	0.115 (7)	0.5
H2BA	−0.013639	0.107213	−0.079432	0.139*	0.5
C3B	−0.167 (2)	0.236 (2)	−0.0844 (13)	0.120 (8)	0.5
H3B	−0.219377	0.206308	−0.154646	0.144*	0.5
C4B	−0.227 (2)	0.335 (2)	−0.0391 (14)	0.122 (7)	0.5
H4B	−0.311513	0.374797	−0.073359	0.146*	0.5
C5B	−0.1391 (13)	0.3653 (16)	0.0656 (11)	0.121 (7)	0.5
H5B	−0.171095	0.428779	0.106286	0.146*	0.5
C6	−0.0073 (3)	0.3099 (3)	0.1166 (3)	0.0697 (9)	
C7	0.0800 (3)	0.3652 (3)	0.2261 (2)	0.0551 (6)	
C8	0.1906 (2)	0.3107 (2)	0.29209 (19)	0.0442 (5)	
C9	0.3034 (3)	0.3711 (2)	0.39527 (19)	0.0423 (5)	
H9	0.265613	0.450631	0.414792	0.051*	
C10	0.3097 (2)	0.28831 (19)	0.49249 (18)	0.0397 (4)	
C11	0.1699 (2)	0.2645 (2)	0.54253 (19)	0.0428 (5)	
C12	0.1814 (3)	0.1897 (2)	0.6451 (2)	0.0487 (5)	
H12A	0.076892	0.146673	0.648431	0.058*	
H12B	0.215222	0.248890	0.708728	0.058*	
C13	0.2968 (3)	0.0896 (2)	0.65146 (19)	0.0472 (5)	
C14	0.4558 (3)	0.1565 (2)	0.6339 (2)	0.0475 (5)	
H14A	0.508114	0.206829	0.700312	0.057*	
H14B	0.522224	0.092238	0.621679	0.057*	
C15	0.4416 (2)	0.2413 (2)	0.53984 (18)	0.0407 (4)	
C16	0.5947 (3)	0.3529 (2)	0.4253 (2)	0.0436 (5)	
C17	0.4688 (3)	0.4055 (2)	0.37333 (19)	0.0433 (5)	
C18	0.4938 (3)	0.4890 (2)	0.2889 (2)	0.0490 (5)	
C19	0.2299 (4)	−0.0175 (2)	0.5635 (2)	0.0593 (7)	
H19A	0.212443	0.018081	0.492353	0.089*	
H19B	0.131409	−0.059562	0.577396	0.089*	
H19C	0.303981	−0.078220	0.565607	0.089*	
C20	0.3193 (4)	0.0330 (3)	0.7645 (2)	0.0671 (7)	
H20A	0.368584	0.098819	0.819591	0.101*	
H20B	0.385352	−0.033648	0.766154	0.101*	
H20C	0.217960	−0.001696	0.779089	0.101*	
C21	0.3116 (5)	0.1391 (4)	0.0592 (3)	0.1013 (13)	
H21A	0.272294	0.077199	−0.002026	0.122*	0.5
H21B	0.398128	0.107737	0.108434	0.122*	0.5
H21C	0.263219	0.099482	−0.012681	0.122*	0.5
H21D	0.372926	0.078161	0.099154	0.122*	0.5
C22A	0.367 (2)	0.2700 (12)	0.0182 (19)	0.168 (9)	0.5
H22A	0.276172	0.306634	−0.018768	0.252*	0.5
H22B	0.436355	0.259743	−0.031876	0.252*	0.5
H22C	0.421703	0.325606	0.079685	0.252*	0.5
C22B	0.426 (2)	0.2569 (18)	0.043 (2)	0.197 (11)	0.5
H22D	0.389262	0.332725	0.067156	0.295*	0.5
H22E	0.431822	0.261267	−0.033255	0.295*	0.5
H22F	0.529782	0.250560	0.085562	0.295*	0.5

### Atomic displacement parameters (Å^2^)

**Table:**

	*U*^11^	*U*^22^	*U*^33^	*U*^12^	*U*^13^	*U*^23^
Cl1	0.0616 (5)	0.0790 (5)	0.1052 (7)	0.0370 (4)	0.0187 (4)	0.0310 (4)
S1	0.0426 (3)	0.0562 (4)	0.0414 (3)	0.0044 (2)	0.0036 (2)	−0.0003 (2)
O1	0.0626 (12)	0.0652 (11)	0.0621 (11)	−0.0073 (9)	0.0108 (9)	0.0075 (9)
O2	0.0534 (11)	0.0619 (10)	0.0564 (10)	0.0198 (8)	0.0023 (8)	−0.0081 (8)
O3	0.0299 (8)	0.0596 (9)	0.0571 (10)	0.0133 (6)	0.0115 (7)	0.0114 (8)
O4	0.0368 (9)	0.0688 (11)	0.0778 (13)	0.0201 (8)	0.0158 (8)	0.0165 (9)
N1	0.0603 (14)	0.0807 (15)	0.0413 (11)	0.0041 (11)	0.0047 (10)	−0.0001 (10)
N2	0.0351 (11)	0.0729 (14)	0.0812 (17)	0.0109 (10)	0.0177 (11)	0.0219 (13)
N3	0.0721 (17)	0.0741 (15)	0.0679 (16)	0.0045 (13)	0.0106 (13)	0.0187 (13)
C1	0.0552 (16)	0.094 (2)	0.0467 (14)	−0.0058 (15)	−0.0051 (12)	0.0159 (14)
C2A	0.071 (7)	0.085 (6)	0.065 (7)	0.012 (5)	−0.001 (4)	0.015 (5)
C3A	0.074 (8)	0.105 (7)	0.052 (6)	0.000 (5)	−0.009 (5)	0.015 (4)
C4A	0.047 (8)	0.134 (10)	0.076 (7)	0.043 (6)	−0.006 (5)	0.036 (7)
C5A	0.059 (7)	0.112 (8)	0.079 (8)	0.036 (6)	0.026 (6)	0.035 (6)
C2B	0.081 (9)	0.194 (14)	0.040 (5)	−0.052 (8)	−0.023 (4)	0.015 (6)
C3B	0.058 (8)	0.23 (2)	0.054 (8)	−0.009 (9)	−0.018 (7)	0.038 (11)
C4B	0.041 (7)	0.179 (19)	0.126 (12)	0.010 (8)	−0.037 (8)	0.087 (10)
C5B	0.048 (7)	0.189 (15)	0.097 (9)	−0.014 (7)	−0.050 (6)	0.066 (9)
C6	0.0370 (13)	0.102 (2)	0.0637 (17)	0.0021 (14)	−0.0051 (12)	0.0342 (17)
C7	0.0351 (12)	0.0692 (15)	0.0622 (15)	0.0106 (11)	0.0081 (11)	0.0204 (13)
C8	0.0310 (10)	0.0559 (12)	0.0456 (12)	0.0085 (9)	0.0051 (8)	0.0101 (10)
C9	0.0327 (10)	0.0438 (10)	0.0507 (12)	0.0099 (8)	0.0058 (9)	0.0021 (9)
C10	0.0324 (10)	0.0442 (10)	0.0425 (11)	0.0077 (8)	0.0062 (8)	−0.0016 (8)
C11	0.0323 (10)	0.0439 (10)	0.0524 (12)	0.0088 (8)	0.0076 (9)	−0.0047 (9)
C12	0.0405 (12)	0.0576 (12)	0.0516 (13)	0.0104 (10)	0.0157 (10)	−0.0011 (11)
C13	0.0430 (12)	0.0556 (12)	0.0462 (12)	0.0131 (10)	0.0117 (10)	0.0061 (10)
C14	0.0392 (12)	0.0566 (12)	0.0482 (12)	0.0140 (10)	0.0066 (9)	0.0068 (10)
C15	0.0308 (10)	0.0479 (10)	0.0446 (11)	0.0079 (8)	0.0087 (8)	−0.0006 (9)
C16	0.0357 (11)	0.0460 (11)	0.0502 (12)	0.0052 (8)	0.0113 (9)	0.0013 (9)
C17	0.0369 (11)	0.0451 (10)	0.0477 (12)	0.0084 (8)	0.0061 (9)	0.0024 (9)
C18	0.0401 (12)	0.0519 (12)	0.0527 (13)	0.0040 (9)	0.0045 (10)	0.0040 (11)
C19	0.0695 (18)	0.0482 (12)	0.0622 (16)	0.0073 (12)	0.0181 (13)	0.0027 (11)
C20	0.0603 (17)	0.0899 (19)	0.0576 (16)	0.0202 (15)	0.0191 (13)	0.0192 (15)
C21	0.081 (3)	0.166 (4)	0.0550 (18)	0.002 (2)	0.0214 (17)	−0.022 (2)
C22A	0.117 (15)	0.214 (15)	0.159 (14)	−0.104 (10)	0.087 (13)	−0.092 (11)
C22B	0.102 (13)	0.29 (2)	0.177 (18)	−0.112 (12)	0.082 (12)	−0.066 (14)

### Geometric parameters (Å, º)

**Table:**

Cl1—C7	1.735 (3)	C9—C10	1.502 (3)
S1—O1	1.4168 (19)	C9—C17	1.512 (3)
S1—O2	1.4287 (18)	C9—H9	0.9800
S1—N1	1.627 (2)	C10—C15	1.341 (3)
S1—C8	1.753 (2)	C10—C11	1.462 (3)
O3—C16	1.364 (3)	C11—C12	1.506 (3)
O3—C15	1.373 (3)	C12—C13	1.538 (3)
O4—C11	1.225 (3)	C12—H12A	0.9700
N1—C1	1.408 (4)	C12—H12B	0.9700
N1—C21	1.461 (5)	C13—C20	1.525 (4)
N2—C16	1.337 (3)	C13—C14	1.525 (3)
N2—H2A	0.92 (3)	C13—C19	1.528 (3)
N2—H2B	0.91 (3)	C14—C15	1.483 (3)
N3—C18	1.139 (3)	C14—H14A	0.9700
C1—C2A	1.391 (5)	C14—H14B	0.9700
C1—C6	1.394 (5)	C16—C17	1.347 (3)
C1—C2B	1.395 (4)	C17—C18	1.415 (3)
C2A—C3A	1.380 (5)	C19—H19A	0.9600
C2A—H2AA	0.9300	C19—H19B	0.9600
C3A—C4A	1.378 (5)	C19—H19C	0.9600
C3A—H3A	0.9300	C20—H20A	0.9600
C4A—C5A	1.381 (5)	C20—H20B	0.9600
C4A—H4A	0.9300	C20—H20C	0.9600
C5A—C6	1.394 (4)	C21—C22B	1.533 (5)
C5A—H5A	0.9300	C21—C22A	1.534 (5)
C2B—C3B	1.380 (5)	C21—H21A	0.9700
C2B—H2BA	0.9300	C21—H21B	0.9700
C3B—C4B	1.377 (5)	C21—H21C	0.9700
C3B—H3B	0.9300	C21—H21D	0.9700
C4B—C5B	1.380 (5)	C22A—H22A	0.9600
C4B—H4B	0.9300	C22A—H22B	0.9600
C5B—C6	1.399 (5)	C22A—H22C	0.9600
C5B—H5B	0.9300	C22B—H22D	0.9600
C6—C7	1.488 (4)	C22B—H22E	0.9600
C7—C8	1.334 (3)	C22B—H22F	0.9600
C8—C9	1.525 (3)		
			
O1—S1—O2	118.26 (12)	C11—C12—C13	113.76 (19)
O1—S1—N1	108.63 (12)	C11—C12—H12A	108.8
O2—S1—N1	107.59 (13)	C13—C12—H12A	108.8
O1—S1—C8	107.85 (12)	C11—C12—H12B	108.8
O2—S1—C8	110.65 (10)	C13—C12—H12B	108.8
N1—S1—C8	102.75 (12)	H12A—C12—H12B	107.7
C16—O3—C15	119.25 (17)	C20—C13—C14	109.1 (2)
C1—N1—C21	120.9 (3)	C20—C13—C19	109.2 (2)
C1—N1—S1	116.6 (2)	C14—C13—C19	111.0 (2)
C21—N1—S1	121.3 (2)	C20—C13—C12	110.0 (2)
C16—N2—H2A	118 (2)	C14—C13—C12	108.06 (19)
C16—N2—H2B	121.8 (19)	C19—C13—C12	109.5 (2)
H2A—N2—H2B	119 (3)	C15—C14—C13	113.40 (18)
C2A—C1—C6	124.9 (6)	C15—C14—H14A	108.9
C6—C1—C2B	113.5 (7)	C13—C14—H14A	108.9
C2A—C1—N1	114.5 (6)	C15—C14—H14B	108.9
C6—C1—N1	120.4 (2)	C13—C14—H14B	108.9
C2B—C1—N1	126.1 (7)	H14A—C14—H14B	107.7
C3A—C2A—C1	119.7 (12)	C10—C15—O3	122.8 (2)
C3A—C2A—H2AA	120.1	C10—C15—C14	125.8 (2)
C1—C2A—H2AA	120.1	O3—C15—C14	111.38 (18)
C4A—C3A—C2A	113.6 (13)	N2—C16—C17	127.6 (2)
C4A—C3A—H3A	123.2	N2—C16—O3	110.3 (2)
C2A—C3A—H3A	123.2	C17—C16—O3	122.1 (2)
C3A—C4A—C5A	128.8 (11)	C16—C17—C18	117.1 (2)
C3A—C4A—H4A	115.6	C16—C17—C9	123.0 (2)
C5A—C4A—H4A	115.6	C18—C17—C9	119.70 (19)
C4A—C5A—C6	116.7 (8)	N3—C18—C17	178.6 (3)
C4A—C5A—H5A	121.7	C13—C19—H19A	109.5
C6—C5A—H5A	121.7	C13—C19—H19B	109.5
C3B—C2B—C1	120.6 (12)	H19A—C19—H19B	109.5
C3B—C2B—H2BA	119.7	C13—C19—H19C	109.5
C1—C2B—H2BA	119.7	H19A—C19—H19C	109.5
C4B—C3B—C2B	129.0 (14)	H19B—C19—H19C	109.5
C4B—C3B—H3B	115.5	C13—C20—H20A	109.5
C2B—C3B—H3B	115.5	C13—C20—H20B	109.5
C3B—C4B—C5B	108.4 (13)	H20A—C20—H20B	109.5
C3B—C4B—H4B	125.8	C13—C20—H20C	109.5
C5B—C4B—H4B	125.8	H20A—C20—H20C	109.5
C4B—C5B—C6	126.5 (11)	H20B—C20—H20C	109.5
C4B—C5B—H5B	116.7	N1—C21—C22B	116.6 (12)
C6—C5B—H5B	116.7	N1—C21—C22A	105.2 (9)
C1—C6—C5A	115.9 (6)	N1—C21—H21A	110.7
C1—C6—C5B	121.9 (7)	C22A—C21—H21A	110.7
C1—C6—C7	119.9 (2)	N1—C21—H21B	110.7
C5A—C6—C7	124.1 (6)	C22A—C21—H21B	110.7
C5B—C6—C7	118.0 (6)	H21A—C21—H21B	108.8
C8—C7—C6	124.5 (3)	N1—C21—H21C	108.1
C8—C7—Cl1	118.5 (2)	C22B—C21—H21C	108.1
C6—C7—Cl1	116.9 (2)	N1—C21—H21D	108.1
C7—C8—C9	127.2 (2)	C22B—C21—H21D	108.1
C7—C8—S1	114.9 (2)	H21C—C21—H21D	107.3
C9—C8—S1	117.90 (15)	C21—C22A—H22A	109.5
C10—C9—C17	109.45 (17)	C21—C22A—H22B	109.5
C10—C9—C8	113.45 (17)	H22A—C22A—H22B	109.5
C17—C9—C8	110.8 (2)	C21—C22A—H22C	109.5
C10—C9—H9	107.6	H22A—C22A—H22C	109.5
C17—C9—H9	107.6	H22B—C22A—H22C	109.5
C8—C9—H9	107.6	C21—C22B—H22D	109.5
C15—C10—C11	118.2 (2)	C21—C22B—H22E	109.5
C15—C10—C9	122.7 (2)	H22D—C22B—H22E	109.5
C11—C10—C9	118.99 (18)	C21—C22B—H22F	109.5
O4—C11—C10	119.4 (2)	H22D—C22B—H22F	109.5
O4—C11—C12	121.9 (2)	H22E—C22B—H22F	109.5
C10—C11—C12	118.57 (19)		
			
O1—S1—N1—C1	63.5 (2)	O1—S1—C8—C9	105.95 (19)
O2—S1—N1—C1	−167.3 (2)	O2—S1—C8—C9	−24.8 (2)
C8—S1—N1—C1	−50.5 (2)	N1—S1—C8—C9	−139.41 (19)
O1—S1—N1—C21	−128.9 (3)	C7—C8—C9—C10	131.5 (3)
O2—S1—N1—C21	0.3 (3)	S1—C8—C9—C10	−50.9 (3)
C8—S1—N1—C21	117.1 (3)	C7—C8—C9—C17	−104.9 (3)
C21—N1—C1—C2A	38.9 (9)	S1—C8—C9—C17	72.7 (2)
S1—N1—C1—C2A	−153.4 (8)	C17—C9—C10—C15	−8.0 (3)
C21—N1—C1—C6	−135.3 (3)	C8—C9—C10—C15	116.3 (2)
S1—N1—C1—C6	32.4 (4)	C17—C9—C10—C11	167.86 (18)
C21—N1—C1—C2B	47.2 (11)	C8—C9—C10—C11	−67.8 (3)
S1—N1—C1—C2B	−145.1 (11)	C15—C10—C11—O4	176.9 (2)
C6—C1—C2A—C3A	−6.0 (19)	C9—C10—C11—O4	0.8 (3)
N1—C1—C2A—C3A	−179.9 (13)	C15—C10—C11—C12	0.6 (3)
C1—C2A—C3A—C4A	0 (3)	C9—C10—C11—C12	−175.43 (18)
C2A—C3A—C4A—C5A	5 (4)	O4—C11—C12—C13	152.7 (2)
C3A—C4A—C5A—C6	−4 (4)	C10—C11—C12—C13	−31.1 (3)
C6—C1—C2B—C3B	4 (2)	C11—C12—C13—C20	171.6 (2)
N1—C1—C2B—C3B	−178.5 (15)	C11—C12—C13—C14	52.6 (3)
C1—C2B—C3B—C4B	−2 (4)	C11—C12—C13—C19	−68.4 (3)
C2B—C3B—C4B—C5B	−2 (4)	C20—C13—C14—C15	−165.8 (2)
C3B—C4B—C5B—C6	3 (4)	C19—C13—C14—C15	73.8 (3)
C2A—C1—C6—C5A	7.0 (12)	C12—C13—C14—C15	−46.2 (3)
N1—C1—C6—C5A	−179.4 (7)	C11—C10—C15—O3	−173.24 (19)
C2B—C1—C6—C5B	−3.1 (14)	C9—C10—C15—O3	2.7 (3)
N1—C1—C6—C5B	179.2 (9)	C11—C10—C15—C14	5.8 (3)
C2A—C1—C6—C7	−168.8 (9)	C9—C10—C15—C14	−178.3 (2)
C2B—C1—C6—C7	−177.4 (10)	C16—O3—C15—C10	3.6 (3)
N1—C1—C6—C7	4.8 (4)	C16—O3—C15—C14	−175.57 (18)
C4A—C5A—C6—C1	−2.2 (19)	C13—C14—C15—C10	19.0 (3)
C4A—C5A—C6—C7	173.3 (14)	C13—C14—C15—O3	−161.91 (19)
C4B—C5B—C6—C1	0 (3)	C15—O3—C16—N2	176.7 (2)
C4B—C5B—C6—C7	174.1 (19)	C15—O3—C16—C17	−3.2 (3)
C1—C6—C7—C8	−16.8 (4)	N2—C16—C17—C18	1.6 (4)
C5A—C6—C7—C8	167.8 (8)	O3—C16—C17—C18	−178.6 (2)
C5B—C6—C7—C8	168.6 (9)	N2—C16—C17—C9	176.8 (2)
C1—C6—C7—Cl1	160.2 (2)	O3—C16—C17—C9	−3.4 (3)
C5A—C6—C7—Cl1	−15.2 (8)	C10—C9—C17—C16	8.4 (3)
C5B—C6—C7—Cl1	−14.4 (9)	C8—C9—C17—C16	−117.4 (2)
C6—C7—C8—C9	168.9 (2)	C10—C9—C17—C18	−176.56 (19)
Cl1—C7—C8—C9	−8.1 (4)	C8—C9—C17—C18	57.6 (3)
C6—C7—C8—S1	−8.7 (4)	C1—N1—C21—C22B	77.0 (11)
Cl1—C7—C8—S1	174.30 (13)	S1—N1—C21—C22B	−90.1 (11)
O1—S1—C8—C7	−76.2 (2)	C1—N1—C21—C22A	56.8 (9)
O2—S1—C8—C7	153.1 (2)	S1—N1—C21—C22A	−110.3 (8)
N1—S1—C8—C7	38.5 (2)		

### Hydrogen-bond geometry (Å, º)

**Table:**

*D*—H···*A*	*D*—H	H···*A*	*D*···*A*	*D*—H···*A*
N2—H2*A*···O4^i^	0.92 (3)	2.03 (3)	2.857 (3)	150 (3)
N2—H2*B*···Cl1^i^	0.91 (3)	2.84 (3)	3.583 (3)	140 (2)
C4*B*—H4*B*···N3^ii^	0.93	2.49	3.381 (18)	160
C19—H19*C*···O3^iii^	0.96	2.56	3.452 (3)	155
C20—H20*C*···O1^iv^	0.96	2.56	3.443 (4)	153

Symmetry codes: (i) *x*+1, *y*, *z*; (ii) −*x*, −*y*+1, −*z*; (iii) −*x*+1, −*y*, −*z*+1; (iv) −*x*, −*y*, −*z*+1.
